# Predictive biomarkers of colon cancer immunotherapy: Present and future

**DOI:** 10.3389/fimmu.2022.1032314

**Published:** 2022-11-22

**Authors:** Wanting Hou, Cheng Yi, Hong Zhu

**Affiliations:** Department of Medical Oncology Cancer Center, West China Hospital, Sichuan University, Sichuan, China

**Keywords:** biomarker, immune checkpoint inhibitor (ICI), colon cancer, predictive, immunotherapy

## Abstract

Immunotherapy has revolutionized colon cancer treatment. Immune checkpoint inhibitors (ICIs) have shown clinical benefits for colon cancer patients, especially those with high microsatellite instability (MSI-H). In 2020, the US Food and Drug Administration (FDA)-approved ICI pembrolizumab as the first-line treatment for metastatic MSI-H colon cancer patients. Additionally, neoadjuvant immunotherapy has presented efficacy in treating early-stage colon cancer patients. Although MSI has been thought of as an effective predictive biomarker for colon cancer immunotherapy, only a small proportion of colon cancer patients were MSI-H, and certain colon cancer patients with MSI-H presented intrinsic or acquired resistance to immunotherapy. Thus, further search for predictive biomarkers to stratify patients is meaningful in colon cancer immunotherapy. Except for MSI, other biomarkers, such as PD-L1 expression level, tumor mutation burden (TMB), tumor-infiltrating lymphocytes (TILs), certain gut microbiota, ctDNA, and circulating immune cells were also proposed to be correlated with patient survival and ICI efficacy in some colon cancer clinical studies. Moreover, developing new diagnostic techniques helps identify accurate predictive biomarkers for colon cancer immunotherapy. In this review, we outline the reported predictive biomarkers in colon cancer immunotherapy and further discuss the prospects of technological changes for biomarker development in colon cancer immunotherapy.

## Introduction

Colon cancer is currently one of the malignant tumors with a high incidence and death rate worldwide (1). Traditionally, the main therapeutic strategies in colon cancer include surgery, chemotherapy, and targeted therapy. Among these therapy methods, surgery is applicable to early-stage patients with lesions confined to the colon, while approximately 20% of colon cancer patients have distant metastases at the time of diagnosis and miss the opportunity for surgery (2). Chemotherapy is the main treatment option for metastatic colon cancer patients. In addition, depending on the RAS gene mutational status and tumor location, corresponding targeted agents, such as bevacizumab or cetuximab, were combined to enhance the anti-tumor effect of chemotherapy agents. Even so, the patients’ prognosis is still dismal (3). Recently, immunotherapy has revolutionized colon cancer treatment. In the clinical study, pembrolizumab, an anti-programmed cell death 1 (PD-1) agent, resulted in significant improvements in progression-free survival (16.5 vs. 8.2 months) and fewer adverse events than chemotherapy as a first-line treatment in patients with microsatellite instability-high (MSI-H) or mismatch repair-deficient (dMMR) metastatic colorectal cancer (mCRC) (4). This promising result led to the US Food and Drug Administration’s (FDA) approval of pembrolizumab as the first-line treatment for MSI-H mCRC patients. Moreover, double ICI combination, anti-cytotoxic T lymphocyte-associated antigen 4 (CTLA-4) agent, and anti-PD-1 agent combination therapy presented promising anti-tumor efficacy in MSI-H/dMMR mCRC (5, 6). Furthermore, neoadjuvant immunotherapy also presented promising efficacy in treating early-stage MSI-H/dMMR colon cancer patients (7).

Currently, MSI-H/dMMR is the only well-recognized biomarker that can be used to guide the immunotherapy of colon cancer. However, the mechanism of why MSI-H/dMMR can be used as a biomarker for colon cancer immunotherapy is still not clarified (8). The possible mechanism was thought to be that MSI-H/dMMR may be correlated with a higher mutational load, which leads to neoantigen formation and activation of the body’s immunity (9, 10). The efficiency of MSI as the biomarker for cancer immunotherapy is relatively low. The reported objective response rate (ORR) in MSI-H/dMMR colon cancer patients varies between 30%-70% (4, 5, 11–14). Additionally, colon cancer patients with MSI-H/dMMR are not in a high proportion of the total number of colon cancer patients. Only approximately 20% of colon cancer patients are MSI-H (8); whereas, in stage IV colon cancer patients, MSI-H is less than 5% (15). In addition, a small percentage of patients with microsatellite stable (MSS) or proficient MMR (pMMR) can benefit from immunotherapy, while a significant proportion of MSI-H/dMMR patients demonstrate intrinsic or acquired resistance to immunotherapy (16–19). These results suggest that more precise biomarkers are needed to stratify colon cancer patients that could benefit from immunotherapy.

Other potential predictive biomarkers which are proposed in colon cancer immunotherapy include PD-L1 expression level, tumor mutation burden (TMB), tumor-infiltrating lymphocytes (TILs), gut microbiota, ctDNA, and circulating immune cells (20–24). In addition, relevant indicators that reflect the tumor microenvironment (TME) have also been proposed for use as biomarkers for immunotherapy in colon cancer (25). Immunoscore and consensus molecular subtypes (CMS) based on immune cells and molecular typing have implications for clinical management and are predictive of prognosis and treatment response in patients with colon cancer (26, 27). New techniques, such as multiplex immunohistochemistry (mIHC) and single-cell RNA sequencing, could provide a more comprehensive evaluation of the TME and genetic heterogeneity in colon cancer. This will help find precise biomarkers for screening and efficacy assessment of colon cancer immunotherapy beneficiary populations (28, 29). In this review, we comprehensively summarize the reported biomarkers of colon cancer immunotherapy and further discuss the prospects of technological changes for biomarker development in colon cancer immunotherapy.

## MSI and dMMR as the predictive biomarker for colon cancer immunotherapy

Microsatellites (MS) are short tandem repeats (STRs) in the human genome that are composed of several short and repetitive DNA sequences. Microsatellite instability (MSI) refers to the failure of the DNA mismatch repair mechanism during the DNA replication process, which results in length changes in MS (8). MSI was first identified in hereditary nonpolyposis colorectal cancer syndrome, known as Lynch syndrome (30). Subsequently, multiple types of malignant tumor patients were found to present with MSI (31–35). MSI is an indicator of tumor prognosis and treatment response (36). According to the status of MMR, tumors can be classified as dMMR and pMMR. IHC is the main method to test the MMR status. Tumors with loss of expression of MMR genes, including MutS homolog 2 (MSH2), MutL homolog 1 (MLH1), MutS homolog 6 (MSH6), or postmeiotic segregation increased by 2 (PMS2), were defined as dMMR; otherwise, they were defined as pMMR. In addition, according to the mutation frequency of MS, tumors can be termed MSI-H, low-frequency MSI (MSI-L), and MSS. Polymerase chain reaction (PCR) is the main method used to assess the frequency of MSI mutations. Due to the respective limitations of these two methods, a combination test of both IHC and PCR is usually required to evaluate the status of MS (37). In general, MSI-H is equivalent to dMMR.

Approximately 20% of colon cancer patients are MSI-H/dMMR (8). Except for approximately 3% of MSI-H/dMMR colon cancer patients who were hereditary with Lynch syndrome, most MSI-H/dMMR colon cancer patients are sporadic. The mechanisms of MSI in hereditary and sporadic MSI-H/dMMR patients are different (8). MSI-H/dMMR colon cancer patients all present unique clinicopathological features that correlate with patient prognosis and treatment response. For example, colon cancer patients with MSI-H/dMMR were reported to have a better prognosis than those with MSI-L/pMMR (38, 39). Colon cancer patients with MSI-H/dMMR did not benefit from fluorouracil-based therapy (40). In particular, there is now considerable clinical evidence that MSI-H/dMMR colon cancer patients present a high ORR to ICIs. The relationship between MSI and colon cancer immunotherapy response was initially found in a phase I clinical trial. In this study, the safety and tolerability of the anti-PD-1 antibody BMS-936558 were evaluated in treatment-refractory solid tumor patients. A post-operative recurrent colon cancer patient who reported a durable complete response after therapy was MSI-H (41). Then, the KEYNOTE-016 trial was conducted to identify the role of MMR status as a biomarker for predicting the clinical benefit of ICI treatment. In this study, progressive metastatic carcinoma patients with or without dMMR were treated with the PD-1 antibody pembrolizumab. In the cohort of patients with dMMR colorectal adenocarcinomas, the reported ORR was 40% versus 0% in the cohort of pMMR patients (11). A similar result was observed in another clinical study, KEYNOTE164. In this study, the antitumor activity of pembrolizumab was tested in previously treated and metastatic MSI-H/dMMR colorectal cancer (CRC) patients. The reported ORR was 33% (14). In addition, in CheckMate 142, another PD-1 agent called nivolumab also induced durable responses and disease control in pretreated dMMR/MSI-H metastatic CRC patients. At a median follow-up of 12.0 months, 31.1% of patients achieved an investigator-assessed objective response (13). Based on these results, the FDA approved ICIs (pembrolizumab and nivolumab) for the treatment of MSI-H/dMMR metastatic CRC patients in 2017. Then, in KEYNOTE-177, the efficacy of pembrolizumab was compared with chemotherapy as first-line therapy for MSI-H/dMMR advanced or metastatic CRC. After a median follow-up of 32.4 months, pembrolizumab therapy demonstrated superiority over chemotherapy in terms of median progression-free survival (16.5 months vs. 8.2 months). ORR was observed in 43.8% of the patients in the pembrolizumab group versus 33.1% in the chemotherapy group (4). This result prompted the FDA to approve pembrolizumab as the first-line treatment for metastatic CRC patients with MSI-H. In addition, in the GERCOR NIPICOL phase II study, an impressive DCR was observed in MSI/dMMR mCRC patients treated with anti-PD-1 inhibitor nivolumab combined with anti-CTLA-4 inhibitor ipilimumab (6). Recently, the preliminary results of nivolumab plus low-dose ipilimumab as the first-line therapy cohort from the CheckMate 142 study were reported. This double ICI combination demonstrated a durable clinical benefit as a first-line treatment for MSI-H/dMMR mCRC. The ORR is 69% in the combination group (5).

However, although MSI-H/dMMR has a promising prospect as the biomarker for colon cancer immunotherapy, the reported ORRs in MSI-H mCRC patients vary from 30%-70% (5, 6, 11–14); this implies that a certain number of MSI-H mCRC patients also do not benefit from immunotherapy. In contrast, a small subset of MSS colon cancer patients responded to immunotherapy (42). The diagnostic mistake caused by the test method is one of the reasons for this phenomenon (18). The current methods could be used for detecting MSI, including IHC, PCR, and next-generation sequencing (NGS). By using IHC to detect the expression of four MMR genes (MLH1, MSH2, MSH6, and PMS2) in the tumor cell nucleus, the presence of one or more negative proteins was defined as dMMR; otherwise, they were defined as pMMR. The advantage of IHC lies in that it is easy to perform and allows direct identification of the MMR gene status. The disadvantages of IHC are its subjectivity and lack of a uniform standard (43). PCR was based on comparing DNA extracted from tumor tissue and normal tissue to detect MSI status. In 1997, the National Cancer Institute (NCI) first formalized the guidelines for PCR testing of MSI, which contained 2 single nucleotide repeat sites (BAT-25 and BAT-26) and 3 dinucleotide repeat sites (D2S123, D5S346, and D17S250) (44). Subsequent studies identified the limitations of this criterion and improved the criteria for PCR detection of MSI. Currently, the five poly-A panel (BAT-25, BAT-26, NR-21, NR-24, and NR-27) is the usually recommended panel for MSI-PCR tests. MSI at more than two loci out of five is defined as MSI-high (MSI-H); MSI at one of five loci is defined as MSI-low (MSI-L); and no instability at any of the loci is defined as MSS (45). PCR is the currently accepted gold standard for the detection of MSI, with high accuracy and standardization. The disadvantage of PCR for MSI detection is that it cannot directly determine abnormal proteins. In addition, considering the difference in MSI incidences among different ethnicities, although there are standardized recommendations, the selection of the appropriate panel for different populations is also essential for the detection of MSI PCR tests (46). NGS is the third alternative method for MSI measurement, which determines MSI by directly measuring the length of altered MS. The NGS method does not require normal tissue as a control, requires lower sample quality, and is more compatible than PCR. NGS can simultaneously provide information on MSI loci, MMR gene status, and information on other gene statuses. In addition, NGS can detect MSI status by peripheral blood samples, unlike traditional PCR methods. However, what limit the clinical promotions and uses of NGS are the high cost, complexity of the data analysis process, and lack of uniform evaluation criteria (47).

Furthermore, reasonable direct evidence for MSI-H/dMMR as a biomarker for immunotherapy is still lacking. Existing studies suggest that the main reason for the response of MSI-H patients to immunotherapy is that MSI may cause the production of new antigens, leading to the recruitment of immune cells and the release of proinflammatory factors. A higher TMB and infiltration of TILs were found in patients with MSI-H/dMMR (48). However, TMB and MSI do not always match perfectly (49). Immunosuppressive cells, such as myeloid-derived suppressor cells (MDSCs) and T-regulatory cells (Tregs), were also found in MSI-H/dMMR patients, which means that a more comprehensive biomarker portfolio is needed for immunotherapy efficacy prediction (50).

## TMB as the predictive biomarker for colon cancer immunotherapy

TMB is another potential effective biomarker in the field of tumor immunotherapy. TMB is usually defined as the total number of somatic mutations detected per million bases (muts/Mb). It was thought that a high TMB (TMB-H) status was related to more tumor neoantigens, and more tumor neoantigens presented on the surface of tumor cells may be recognized by immune cells and activate the body immune system to kill the tumor (51). In KEYNOTE-158, pembrolizumab was tested in several types of advanced solid tumor patients but did not include colon cancer patients. The results show that patients with TMB-H status (TMB≥10 muts/Mb) presented higher objective responses to pembrolizumab. The ORRs were reported to be 29% in the TMB-H group versus 6% in the non-TMB-H group (52). Based on this result, in June 2020, the FDA approved the use of pembrolizumab for unresectable or metastatic solid tumor patients with TMB-H status. With a cutoff value of 10 muts/Mb, higher response rates to immunotherapy were confirmed in melanoma and non-small cell lung cancer (NSCLC) patients with TMB-H (53, 54).

In colon cancer, TMB was found to be potentially correlated with patient survival. It was reported that colon cancer patients with high TMB (TMB≥8 muts/Mb) presented longer OS than those with low TMB (55). Additionally, TMB was reported as an additional predictive biomarker for MSI in metastatic CRC. Among MSI-H mCRC patients, patients with TMB-H (the TMB cutoff point was defined between 37 and 41 mutations/Mb) have shown a better prognosis than those with TMB-L after receiving immunotherapy (18). However, the independent application of TMB for the prediction of immunotherapy response in colon cancer is still controversial. In the KEYNOTE 177 trial, the limits of TMB as a predictor of the response of CRC to anti-PD1 immunotherapy were observed (56). However, in the Canadian cancer trials group CO.26 study, elevated plasma TMB levels (≥28 muts/Mb) showed a predictable response to anti-PD-L1 agent durvalumab and anti-CTLA4 agent tremelimumab combination therapy in MSS colon cancer patients (57).

One of the greatest obstacles causing this controversy is the difficulty in defining the TMB cutoff value. Nonetheless, the cutoff of 10 muts/Mb presented a relatively good sensitivity in the prediction of immunotherapy in NSCLC and melanoma, but this cutoff value cannot be generalized for different tumor types (58). Currently, there is no uniform TMB cutoff value for colon cancer. In a recently reported study, TMB≥16 mut/Mb was proposed as the optimal threshold for ICI atezolizumab monotherapy in advanced solid tumor types. In patients with TMB ≥16 mut/Mb, durable clinical activity was observed, and particularly high response rates (70%) were reported in CRC patients, including both MSI-H and MSI-L (59). Nonetheless, this finding still awaits further validation by prospective studies. In addition, TMB is proposed as a predictive biomarker for immunotherapy because it may represent a useful estimation of tumor neoantigens. However, not all neoantigens presented on the cell surface are immunogenic. Only mutations resulting in higher ‘quality’ antigens can induce an antitumor immune response (60, 61). This may explain why TMB cannot be used as an independent marker of the effectiveness of tumor immunotherapy. Furthermore, the testing method hinders the clinical application of TMB. TMB was initially performed using whole-exome sequencing (WES), and this technology is complex and costly. Currently, NGS has been used in the clinic as a substitute for WES; however, there are different algorithms for WES-based and NGS-panel-based methods. In addition, the advantage of TMB as the biomarker for immunotherapy is that TMB can be not only obtained from tumor tissue but also detected by peripheral blood. Therefore, in some studies, tissue TMB was substituted with plasma TMB when unavailable. But there are differences in the criteria for assessing TMB in blood samples versus tissue samples, which cause inconsistencies among different studies and interfere with TMB standardization (62).

In summary, although several pieces of evidence indicate that TMB cannot be used as an independent predictive biomarker in colon cancer immunotherapy, it is still valuable in immunotherapy efficacy prediction, especially when sufficient evidence is obtained for a valid TMB cutoff value in colon cancer. TMB can be used as an important complementary biomarker, such as when combined with MSI, and for identifying other significant gene mutations; it was found, that some MSS colon cancer patients with high TMB had polymerase epsilon (POLE) mutation, and they responded well to immunotherapy (63).

## The value of molecular subtype and consensus molecular subtype as predictive biomarkers in colon cancer immunotherapy

Colon cancer is a heterogeneous disease. Colon cancer patients usually present different molecular subtypes. Some molecular subtypes, such as RAS and BRAF mutations, have already been used to guide the treatment and prognostic assessment of colon cancer (64). Among these molecular subtypes, the encoded DNA POLE and delta 1 (POLD1) mutation has attracted much attention due to its potential association with immunotherapy response. POLE/POLD1 play an important role in proofreading and ensuring the fidelity of DNA replication. The somatic or germline mutations in POLE and POLD1 lead to defects in DNA repair and, consequently, to tumorigenesis (65). In colon cancer, about 7.4% of patients harbor POLE or POLD1 mutations, and most of this population was MSS or MSI-L (66). In 2019, Wang and his colleagues, through analyzing medical data of 47,721 patients with various cancer types with POLE/POLD1 mutations, proposed that POLE/POLD1 mutations are promising potential predictive biomarkers for positive ICI outcomes (66). However, in a previous clinical study, which enrolled three CRC patients with POLE mutations, three of the patients did not show an achieved response to anti-PD-L1 inhibitor avelumab (67). In a recent study, where anti-PD-L1 inhibitor durvalumab monotherapy was used to treat previously treated MSI-H/dMMR or POLE-mutated mCRC patients, the results showed that POLE mutation mCRC patients had a clinical response to durvalumab, only those with exonuclease domain mutation (68). Furthermore, there is a limited role of other molecular mutations as biomarkers in predicting the response of colon cancer to immunotherapy; this includes KRAS mutation, a common molecular subtype in colon cancer. Although KRAS mutation is proposed to modulate tumor immunity (69), its biomarker value in colon cancer immunotherapy was found to be weak. According to Lal et al., KRAS mutation is proposed to be associated with suppressed cytotoxic immunity in CRC, and the extent of the effect is modulated by consensus molecular subtype (CMS) (70).

The CRC Subtyping Consortium, based on the gene expression of the tumor, proposed four CMSs for CRC using transcriptomics in 2015. The CMS classification included CMS1, CMS2, CMS3, and CMS4. CMS1 is categorized as MSI immune and presents with strong immune activation; CMS2 is categorized as canonical and is characterized by chromosomal instability as well as WNT and MYC signaling activation; CMS3 is categorized as metabolic and is associated with metabolic dysregulation; CMS4 is categorized as mesenchymal and is associated with prominent transforming growth factor β (TGF-β) activation, stromal invasion, and angiogenesis (71). CMS classification can be used in guiding colon cancer treatment strategies and predicting patient prognosis (72–74). The correlation between CMS and tumor immune characteristics has been proposed in several studies (27, 75). In 2016, Becht E et al. integrated the CMS classification with the TME of CRC. Their results show that the good prognosis of CMS1 is related to the overexpression of cytotoxic lymphocytes. In contrast, the poor prognosis of CMS4 is related to a high density of fibroblasts, which produce chemokines and cytokines, resulting in inflammatory and immunosuppressive TMEs. The CMS2 and CMS3 groups presented an intermediate prognosis, exhibiting low immune and inflammatory signatures (75). In a recent study, Hu et al, based on data from multiple databases and using algorithms, further analyzed the molecular characteristics of colon cancer CMS and their immunotherapy responses. Their results indicate that CMS1 patients present a higher positive response to immunotherapy among the four CMS subtypes due to immune infiltration and activation. TILs were significantly higher in the CMS1 subtype than in the other three subtypes. In contrast, CMS4 patients may not respond well to immunotherapy, due to the high Treg and NK-cell infiltration found in the CMS4 subtype (27). Meanwhile, in Chida K and his colleagues’ study, transcriptomic profiling for MSI-H/dMMR gastrointestinal tumors was performed to determine the predictors of response to PD-1 blockade. The results show among 13 CRC patients, the reported ORR for CMS1 was 100%, for CMS4 was 16.7%, but for CMS2 and CMS3 all were 0%. This study indicates that CMS classification may serve as a predictive biomarker for colon cancer immunotherapy (76).

However, existing CMS classification still has certain limits. CMS classification relies on transcriptome analysis of the entire tumor, which has inherent limitations such as stromal confounding and the presence of varied cell-type mixtures. Moreover, the differences in cancer cells and other stromal cells (e.g., immune cells, fibroblasts, and vascular cells) are masked and indistinguishable (77, 78). There is transcriptomic intratumor heterogeneity in CMS classification, which may impact its accuracy (79). To solve this, Joanito et al, using single-cell and bulk transcriptome sequencing, identified two epithelial tumor cells and refined the CMS classification of colon cancer. The refined CMS classification includes intrinsic epithelial subtype, MSI status, and fibrosis. By this classification, a specific subtype of MSS was identified. They proved that despite a lower TMB, iCMS3_MSS tumors are more similar to MSI-H colon cancers, and this refined classification may provide new clues for screening the population benefiting from immunotherapy in colon cancer (80). Recently, Khaliq et al. also refined CRC classification and clinical stratification through a single-cell atlas; they proved that distinct cancer-associated fibroblasts (CAFs) and tumor-associated macrophages (TAMs) are sufficient to explain CMS predictive ability and, based on these cellular phenotypes, could stratify CRC patient prognosis with greater precision (81).

In conclusion, existing molecular subtypes and CMS may not be suitable for stratifying colon cancer patients for immunotherapy. The molecular subtypes and CMS help define the molecular and immunological characteristics of colon cancer, which contribute to the precise therapy of colon cancer. As research progresses, the understanding of the molecules subtype of colon cancer patients continues to improve and a more precise molecular subtype of colon cancer may be recognized, and a more precise CMS classification may be refined, which will further contribute to colon cancer immunotherapy.

## TILs, immunescore, and PD-L1 expression as predictive biomarkers for colon cancer immunotherapy

TME plays an essential role in tumorigenesis, development, and immune escape (82). Including immune cells, other components of the TME can influence the immune state and response to immunotherapy in tumors (83). TILs are core components of immune cells involved in tumor immunity. TIL is a global term for a variety of lymphocytes in the TME, including T cells, B cells, and NK cells. Several studies have proven that TILs play prominent roles in malignant tumor development and progression and have been proposed as predictive biomarkers for patient prognosis (84–86). The relationship between TILs and colon cancer patient prognosis was first reported in 1998. In this study, CD8+ T cells infiltrated within cancer cell nests were observed to be a prognostic factor in human CRC (87). A series of studies then reported the role of TILs in the prognosis of patients with colon cancer (88–91).

In several clinical trials of colon cancer immunotherapy, TILs showed potential in being used as predictive biomarkers for immune response. In an analysis study of the KEYNOTE 177 trial, colon cancer patients’ response to immunotherapy was found to be not associated with TMB, but rather with TILs. Immunotherapy-responsive CRC patients were found rich in CD-8+PD-1+ T cells (56). In Loupakis F et al’ study, they proposed that there was a significant positive correlation between high TMB and the number of TILs in the ICI-responsive MSI-H mCRC patients (92). In the pilot clinical trial of perioperative durvalumab combined with tremelimumab for treating resectable CRC liver metastases, the treatment induced activation of CD8+ and CD4+ T cells, and an increase in B-cell density was correlated with patients’ prolonged relapse-free survival (93). In the study of neoadjuvant immunotherapy for early-stage colon cancer patients, CD8+PD-1+ T cell infiltration was a predictive biomarker of response in pMMR patients (7). For MSS mCRC patients, higher CD8+ TIL density at baseline was associated with a greater likelihood of benefit from immunotherapy treatment and activated TILs are considered as the biomarker of effective immune induction (94). And according to Kuang C et al’s study, immune modulation may result from treatment with azacitidine, chemotherapy refractory mCRC patients with higher CD8+TIL density at baseline are more likely to benefit from the combination therapy of pembrolizumab and azacitidine combination (95). In addition to CD8+T cells, the role of CD3+T cells as the predictive biomarker in colon cancer immunotherapy has also been reported in some studies (96, 97). In Turksma et al’s study, they found that high numbers of pre-existing stromal CD3+ T cells are own positive predictive value in adjuvant immunotherapy treatment for MSS colon cancer patients (96). In Chakrabarti S. et al’s study, higher CD3+ and CD8+ T-cell densities were associated with higher ORR in dMMR mCRC patients treated with pembrolizumab (97).

CD3+ T cells and CD8+ T cells are two important types of TILs that represent the total T cells and cytotoxic T cell subsets, respectively. In 2018, the Society for Immunotherapy of Cancer proposed using Immunoscore (IS) to estimate the risk of recurrence in colon cancer patients, and their findings proved the powerful role of IS in CRC recurrence risk assessment (98). IS is based on the quantification of CD3+ T cells and CD8+ T cells at the tumor center and at the invasive margin using IHC. A scoring system ranging from IS0 (I0) to IS4 (I4) and high IS was associated with prolonged survival in CRC (99). In Mlecnik et al’s study, IS is proposed to play a bigger role in predicting CRC patient survival than MSI (100). However, IS as the predictive biomarker for colon cancer immunotherapy has certain limitations. First, IS assays are mainly performed by IHC, which is a semiquantitative test, and the results are susceptible to subjectivity. Second, the IS test requires simultaneous assessment of lymphocytes in the center and margin of the tumor, which is difficult to achieve in metastatic tumors. In addition, intratumoral heterogeneity can also affect the accuracy of IS. For example, heterogeneity of T cells was observed in the primary tumor and hepatic metastases of CRC patients (97). Finally, current studies have shown that the effectiveness of tumor immunotherapy is influenced by the immune landscape rather than by a single immune cell (63, 101–103). Except for CD8+T cells, other immune cells, such as Treg cells, NK cells, DC cells, and B cells, are also closely related to the immune response of tumors (104, 105). Recently, a special lymph node structure, tertiary lymphoid structure (TLS), was also proposed as a biomarker in cancer prognosis and response to immunotherapy (106). The effect of TLS on the prognosis of colon cancer has now been demonstrated in several studies (107–110). But the role of TLS in colon cancer immunotherapy still needs to be verified. In addition, the phenotypic profiles and subsets of TILs were also found to affect the patient’s response to ICI and have the potential to be biomarkers of immunotherapy (96, 111–113). For example, a CD39 subgroup of CD8+ T cells was reported in colorectal and lung tumors, the absence of CD39 in CD8+ TILs causes them to act as bystanders that lack an immune response (112). A similar phenomenon has also been observed in B cells; it was found that B cells with CD86 expression were enriched in tumors with increased numbers of TLSs, induced specific T-cell responses, and enhanced the antitumor effect of ICI (114). Epigenetic alterations of TILs, such as DNA methylation, are also involved in the colon cancer immune response. According to Zou et al’s study, the DNA methylation-based signature of CD8+ TILs was related to the immune response and prognosis of CRC patients (22). Thus, further screening TIL subgroups and studying the immune landscape of colon cancer are key to improving the accuracy of screening for beneficial ICI populations.

PD-L1 is another important indicator for TME. Tumor cells induce tumor immune escape by upregulating PD-L1 expression, which binds to PD-1 on the surface of T cells, causing T-cell deactivation. ICIs can reactivate the body’s antitumor immunity by blocking the binding of PD-1 and PD-L1 (115). Thus, in theory, the higher level of PD-L1 expression in tumor tissues, the better the response to ICI treatment. PD-L1 expression is postulated as a predictive biomarker of immunotherapy response in some solid tumors, such as NSCLC, melanoma, and renal cell cancer (116–118). Positive PD-L1 expression (with a cut-off value of 10%) is reported in more than half of colon cancer patients (119, 120). Although a high PD-L1 expression is associated with a better prognosis in colon cancer patients (20, 121–123). The current clinical data suggest that PD-L1 expression alone cannot be used to precisely predict immunotherapy response in colon cancer (**Table 1**). Several factors limit PD-L1 expression as a biomarker for colon cancer immunotherapy response: First, there is intratumoral heterogeneity of PD-L1 expression (135), which makes assessing tumor PD-L1 expression level. Second, PD-L1 expression is dynamic, and treatment modalities can affect the expression level of PD-L1. PD-L1 expression varies widely between tumor types and presents a significant intrapatient heterogeneity with a frequent discordance between primary tumors and metastases. Third, the test method also affects the assessment of PD-L1 expression. IHC is now widely used in clinical practice to detect PD-L1 expression in tumor tissues. However, this method is difficult to quantify, the consistency of detecting PD-L1 expression levels between different platforms is poor, and there is still lack of a standardized testing criteria (136). Lastly, tumor cells and immune cells can both express PD-L1. Thus, the predictive effects of PD-L1 expression by tumor cells and PD-L1 expression by lymphocytes on immunotherapy need to be clarified separately. This is illustrated when PD-L1-expressing tumor cells were reported to be a marker of poor prognosis; in contrast, PD-L1-expressing TILs were a marker of good prognosis (137).

**Table 1:** Summary of biomarkers for colon cancer immunotherapy in reported clinical trials.

Biomarker	Tumor type	Patients’ number	Immunotherapy agent	Association with clinical outcome	Tissue type for biomarker assessment	NCT	Ref
MSI	MSI-H-dMMR mCRC	307	pembrolizumab(anti-PD-1)	dMMR/MSI-H was positive with patients’ clinical outcome	tumor tissue	NCT02563002 (KEYNOTE-177)	(4)
MSI	MSI-H/dMMR mCRC	57	nivolumab(anti-PD-1)+ipilimumab(anti-CTLA-4)	dMMR/MSI-H was positive with patients’ clinical outcome	tumor tissue	NCT03350126	(6)
MSI	advanced dMMR solid tumors	86	pembrolizumab(anti-PD-1)	dMMR/MSI-H was positive with patients’ clinical outcome	tumor tissue and blood	NCT01876511 (KEYNOTE-016)	(11)
MSI	MSI-H/dMMR mCRC	124	pembrolizumab(anti-PD-1)	dMMR/MSI-H was positive with patients’ clinical outcome	tumor tissue	NCT02460198 (Keynote164)	(14)
MSI	dMMR/MSI-H CRC	119	nivolumab(anti-PD-1)+low-dose ipilimumab(anti-CTLA-4)	dMMR/MSI-H was positive with patients’ clinical outcome	tumor tissue	NCT02060188 (CheckMate 142)	(5)
TMB	MSI-H mCRC	22	PD-1/PD-L1 inhibitors	The optimal predictive cut-point for TMB was estimated between 37 and 41 mutations/Mb.	tumor tissue	NA	(18)
TMB/TIL	CRC	29	pembrolizumab(anti-PD-1)/nivolumab(anti-PD-1)	Patients’ response to immunotherapy not associated with TMB, but with TILs.	tumor tissue	NCT02563002 (Keynote177)	(56)
bTMB	advanced CRC	179	durvalumab(anti-PD-L1)+tremelimumab(anti-CTLA-4)	Patients who were MSS with plasma TMB of 28 variants per megabase or more had the greatest OS benefit.	blood	NCT02870920	(57)
MSI/POLE mutation	MSI-H/POLE mutation mCRC	33	avelumab(anti-PD-L1)	Avelumab displayed antitumor activity with manageable toxicity in patients with previously treated mCRC harboring dMMR/MSI-H. Further clinical studies with larger sample sizes are necessary to evaluate the activity of ICIs and its association with sites in POLE-mutated CRC.	tumor tissue	NCT0315-0706	(67)
MSI-H/dMMR or POLE EDM	previously treated MSI-H/dMMR or POLE-mutated metastatic or unresectable CRC	33	durvalumab(anti-PD-L1)	Durvalumab showed promising clinical activity with encouraging response rates and satisfactory survival outcomes in mCRC patients with MSI-H/dMMR or POLE exonuclease domain mutation (EDM). In patients with POLE-mutated mCRC, clinical response to durvalumab may be restricted to those with EDM.	tumor tissue	NCT03435107	(68)
CMS	MSI-H/dMMR gastrointestinal tumors	CRC (n=13)	anti-PD-1 inhibitor	The ORR was 100%,0%,0%,and 16.7% for CMS1, CMS2, CMS3, and CMS4, respectively. Several transcriptomic features,including CMS classification and related genes, were associated with response to PD-1 blockade in MSI-H/dMMR gastrointestinal tumors.	tumor tissue	NA	(76)
TIL	early-stage colon cancer	40	nivolumab(anti-PD-1)+ipilimumab(anti-CTLA-4)	CD8+PD-1+ T cell infiltration was predictive of response in pMMR tumors.	tumor tissue	NCT03026140	(7)
TIL and TMB	MSI-H mCRC	85	ICI	A significant correlation between higher TMB and increased number of TILs was shown. A significantly higher activity and better PFS and OS with ICI in MSI-H mCRC were reported in cases with high number of TILs.	tumor tissue	NA	(92)
TIL	resectable pMMR mCRC	24	perioperative durvalumab(anti-PDL1)+tremelimumab(anti-CTLA-4)	An increase in B-cell transcriptome signature and B cell density was present in post-treatment samples from patients with prolonged RFS.	tumor tissue	NCT02754856	(93)
TIL	MSS mCRC	29	durvalumab(anti-PD-L1) + trametinib (MEKi)	The response rate in the first stage of the study did not meet efficacy criteria to proceed to the second stage. TIL was related with clinical outcome.	tumor tissue	NCT03428126	(94)
TIL	chemotherapy refractory mCRC	30	pembrolizumab(anti-PD-1)+azacitidine(DNA methyltransferase inhibitor)	Higher CD8+ TIL density at baseline was associated with greater likelihood of benefit from treatment.	tumor tissue	NCT02260440	(95)
TIL	MSS colon cancer	106	adjuvant active specific immunotherapy(ASI)	High numbers of pre-existing stromal CD3 positive T cells are of positive predictive value in adjuvant ASI treatment.	tumor tissue	NA	(96)
CPM score (composite PD-L1 and mucin)	advanced mCRC	26	pembrolizumab(anti-PD-1)	The CPM score discriminated patients who exhibited clinical benefit from those patients with progressive disease.	tumor tissue	NCT01876511	(124)
gut microbiome	advanced-stage GI cancer	CRC (n=19)	anti–PD-1/PD-L1 immunotherapy	An elevation of the Prevotella/Bacteroides ratio in patients, with a preferred response to anti–PD-1/PD-L1 treatment.	fecal sample	NA	(125)
gut microbiome	mCRC(97.4% MSS)	33	regorafenib+toripalimab(anti-PD-1)	Gut microbiome analysis of the baseline fecal samples shows significantly increased relative abundance and positive detection rate of Fusobacterium in non-responders than responders.	fecal sample	NA	(126)
gut microbiome	RAS wild‐type mCRC	14	cetuximab + avelumab (anti-PD-L1)	Agathobacter M104/1 and Blautia SR1/5 expression were associated with PFS.	fecal sample	NCT04561336	(127)
ctDNA	Refractory MSS mCRC	18	regorafenib+nivolumab/pembrolizumab	ctDNA may represent a powerful tool for predicting early therapeutic efficacy of immunotherapy in the MSS CRC population.	blood	NA	(128)
ctDNA/NLR	RAS wild type mCRC	77	cetuximab+avelumab(anti-PD-L1)	Plasma ctDNA analysis before treatment may allow selection of patients who could benefit. Baseline NLR <3 significantly correlated with improved survival and may represent a potential predictive biomarker of cetuximab plus avelumab rechallenge activity in ctDNA RAS/BRAF WT patients.	blood	NCT04561336	(129)
circulating immune cells	refractory pMMR mCRC	24	durvalumab(anti-PD-L1)+tremelimumab(anti-CTLA4)+concurrent radiotherapy	Increased circulating CD8+ T lymphocyte activation, differentiation, and proliferation in patients with objective response	blood	NCT03122509	(130)
circulating immune cells	MSS mCRC	10	mFOLFOX6+bevacizumab+CEA-targeted vaccine + avelumab(anti-PD-L1)(SOC+IO)	SOC+IO generated multifunctional MUC1- and brachyury-specific CD4+/CD8+ T cells despite concurrent chemotherapy.	blood	NCT03050814	(131)
circulating immune cells	dMMR/MSI-H CRC	41	anti-PD-1 inhibitor(nivolumab, pembrolizumab,triprizumab, toripalimab, and camrelizumab)	The ratio of CD4+/CD8+ and the frequency of CD4+ Tcell might be crucial independent biomarkers within dMMR mCRC to better identify patients for anti-PD-1 immunotherapy.	blood	NA	(132)
circulating immune cells	mCRC	24	pembrolizumab(anti-PD-1)+modified FOLFOX6	Baseline levels and changes in circulating MDSC and Treg subsets are not associated with RECIST response or mPFS.	tumor tissue and blood	NCT02375672	(133)
NLR	unresectable CEA+ liver mCRC	6	CART	NLR variations and associated cytokine changes may be useful surrogates of response to CAR-T.	blood	NCT01373047	(134)

CRC, colorectal cancer; MSI, microsatellite instability; TMB, total mutation burden; TILs, tumor-infiltrating lymphocytes; CMS, consensus molecular subtype; PD-L1, programmed cell death-ligand 1; CTLA4, Cytotoxic T Lymphocyte antigen 4; ctDNA, circulating tumor DNA; NLR, neutrophil-lymphocyte ratio; CART, Chimeric antigen receptor T cell therapy.

NA, not available.

Nonetheless, PD-L1 expression still has value in immunotherapy for colon cancer patients. The PD-L1 expression level is an important indicator of the immune status of cancer patients (138–140), and the immune status indicates the tumor response to immunotherapy. PD-L1 combined with other immune indicators demonstrated a promising predictive role in colon cancer immunotherapy. Such as, Llosa et al. proposed the incorporation of histopathologic characteristics (percentage of extracellular mucin) and PD-L1 expression at the invasive front to generate a composite score (CPM score). The CPM score has the potential of discriminating mCRC patients who exhibited clinical benefits from pembrolizumab (124). Additionally, using multiplex immunohistochemistry (mIHC), multiple immune indicators combined with PD-L1 expression can be analyzed simultaneously as well as report TME in various solid tumors, including colon cancer (28, 141, 142).

Therefore, TME immune landscape is significantly related to tumor immunotherapy response. It is not sufficient to evaluate tumor response to immunotherapy by a single index only, such as PD-L1 expression or the number of TILs. A more comprehensively quantified TME immune landscape is necessary for the prediction of colon cancer immunotherapy response.

## Other gene signatures of the TME as predictive biomarkers for colon cancer immunotherapy

With the development of gene sequencing technology, several TME-related gene signatures have been proposed as predictive biomarkers for colon cancer immunotherapy. Previously, Ravensbergen et al, using bioinformatics approaches, proved that combined assessment of the tumor-stroma ratio and TILs could be used as a response prediction biomarker of ICI therapy in colon cancer (143). This result reveals the role of the tumor stoma in the response to tumor immunotherapy in patients with colon cancer. CAFs are the main cell type within the tumor stroma, and they are also thought to be an available indicator for assessing the response to immunotherapy. CAFs can interact with tumor cells and TILs *via* the secretion of various cytokines and chemokines, shaping an immunosuppressive TME and helping tumor cell immune evasion. In addition, CAFs play a significant role in constituting the inflammatory TME of colon cancer (144). Some studies have proven that CAF-derived gene signatures can determine prognosis in colon cancer patients (29, 145, 146). In the area of immunotherapy, it was proven that CAFs promote the upregulation of PD-L1 expression in CRC (147). CAFs have an impact on the prognosis of CRC patients by inhibiting the immune response; thus, patients with higher CAF levels were more prone to be unresponsive to immunotherapy (29, 148). Additionally, among the CMSs, CMS4 is typically characterized by infiltration of adjacent tumor tissues by CAFs and transforming growth factor β (TGF-β) signaling activation, and this subtype presented insensitivity to immunotherapy. Recently, the refinement of CMS through single-cell characterization based on specific CAF subtypes presented the potential role of identifying immunotherapy responses in CRC patients (81). Thus, further study of CAF gene signatures may contribute to the precise stratification of immunotherapy efficacy for colon cancer.

TME metabolic characteristics also influence patients’ response to immunotherapy. Hypoxia is one of the metabolic characteristics of the tumor TME. Hypoxia can play an essential role not only in tumor proliferation, apoptosis, angiogenesis, invasion, and metastasis but also in immune evasion. Hypoxia and the related acidic TME greatly impair the functions of TILs, while alleviating hypoxia could improve the efficacy of ICIs (149). Transcriptomic profiling of MSI-H/dMMR gastrointestinal tumors showed that hypoxia-related signaling pathways were upregulated in ICI nonresponders (76). Recently, several studies proposed that hypoxia-related genes can be classified as predicting immune cell infiltration and prognosis of colon cancer patients (25, 28, 150). These genes provide potential therapeutic targets for immunotherapy as well as prognostic biomarkers for colon cancer patients. In addition to hypoxia, ferroptosis-related gene signatures are another study hot spot in the TME-related prognostic assessment of immunotherapy for colon cancer. Several studies support that ferroptosis plays a vital role in tumor immunotherapy and TME regulation, and ferroptosis-related gene signatures were proposed as potential targets for tumor immunotherapy and patient prognosis (151–153). In recent studies, several ferroptosis-related gene signatures were proposed for the prediction of prognosis and immunotherapy response in colon cancer patients (154–156). These findings further confirm the relevance of ferroptosis to the immune microenvironment and prognosis of colon cancer. Therefore, further understanding of the metabolic characteristics of the TME and the search for metabolism-related gene signatures are valuable for the identification of new biomarkers of colon cancer immunotherapy.

Furthermore, the inflammatory microenvironment of colon cancer induces immune-related genetic alterations, and inflammatory-related genes affect the response of patients to immunotherapy. Wang et al. explored the relationship between inflammation-related genes and the immune TME in CRC. Eight prognostic genes (CX3CL1, CCL22, SERPINE1, LTB4R, XCL1, GAL, TIMP1, ADIPOQ, and CRH) were identified and used to construct a risk-scoring model. The results of this study show that the inflammatory response has a direct impact on CRC patient prognosis and immune infiltration. Thus, further classifying inflammatory response-related genes may help find predictive biomarkers for immunotherapy in colon cancer (157).

Through analysis by transgenomic techniques, some TME-related gene signatures were proposed for use as biomarkers for colon cancer immunotherapy. However, most of these studies were derived from bioinformatic analyses of databases; further validation of these genes in large prospective clinical studies is necessary.

## The potential role of certain gut microbiota as the predictive biomarker for colon cancer immunotherapy

The gut microbiota is another hot topic in the current field of immunotherapy. Several studies have proposed that gut microbiota are involved in tumor formation and progression and correlate with patient therapy response in solid cancers (158–164). It has been established that tumor patients have distinct microbiota compared with healthy subjects (165, 166). In addition, compared to patients that did not respond to immunotherapy, a unique intestinal microbiome was found in cancer patients that did respond to immunotherapy (167). Increasing evidence indicates that transplanting the gut microbiome of immunotherapy responders can activate immune cells and make immunotherapy nonresponders respond to immunotherapy (168–171). Therefore, the gut microbiome could be a promising therapeutic target as well as a predictive biomarker in cancer immunotherapy.

Colon cancer presents with an altered state of gut microbiota, which is known as dysbiosis (165). The gut microbiome plays a significant role in the formation of the inflammatory microenvironment during the development of colon cancer. Gut microbes can interact with TILs and influence the tumor immune microenvironment and host sensitivity in favor of immunotherapy in colon cancer (172–174). Recently, certain gut microbes have been proposed as promising predictive biomarkers of colon cancer immunotherapy (23). In 2020, Peng and his colleagues recruited advanced-stage GI cancer patients receiving anti-PD-1/PD-L1 treatment and collected their fecal samples. By comparing the gut microbes of patients before and after treatment, they found an elevation of the Prevotella/Bacteroides ratio in patients with a preferred response to immunotherapy (175). In another phase Ib/II clinical trial of regorafenib plus toripalimab treatment for mCRC, gut microbiome analysis presented a significantly increased relative abundance and positive detection rate of Fusobacterium in nonresponders compared to responders (125). In a recent clinical study, Agathobacter and Blautia species were proposed as potential biomarkers of outcome in mCRC and NSCLC patients treated with cetuximab and avelumab (126). However, the evidence related to the role of gut microbiome as a prognostic marker of colon cancer immunotherapy is still lacking, and further investigations are still required to consider the gut microbiome as a predictive biomarker for the immunotherapy response in colon cancer.

The testing method of gut microbiome detection also needs to be optimized and unified. Current methods for testing the gut microbiome are mainly stool-based genetic tests. The two commonly used methods are PCR-based 16S amplicon sequencing and macrogenome sequencing. PCR-based sequencing of 16S amplicons is relatively less costly, however this method is limited to the genus level and can easily miss microbiomes with low expression levels. In contrast, macrogenomic sequencing has several potential advantages over 16S amplicon sequencing. Macrogenomic sequencing can extend gut microbiome taxonomic resolution to the species level and can also provide information on metabolic pathways of the microbiome. However, the cost of macrogenomic sequencing is relatively high, and the interpretation of analysis results is complex (127). Measurement differences may exist between the two methods due to differences in stool sample collection, storage, and handling, as well as nucleic acid extraction protocols and data analysis methods. Therefore, to make better use of gut microbiota as the predictive biomarker for immunotherapy of colon cancer, it is also necessary to further optimize the testing methods and standardize the testing criteria.

## Peripheral blood biomarkers in colon cancer immunotherapy

CtDNA is the most used peripheral blood biomarker. Previously, ctDNA already presented high sensitivity in colon cancer early diagnosis, recurrence detection, and treatment outcome prediction (176, 177). CtDNA is derived from apoptotic and necrotic tumor cells that release their fragmented DNA into circulation. Information on genetic variation could be detected through ctDNA test (178). In 2017, Cabel et al. proposed a proof-of-concept study, they enrolled patients with NSCLC, uveal melanoma, or MSI CRC who were treated by nivolumab or pembrolizumab monotherapy, their results demonstrated that quantitative ctDNA monitoring can be used as a valuable tool to assess tumor patients’ response to anti-PD-1 agents (179). Several studies also focused on the predictive value of ctDNA in colon cancer immunotherapy. Wang et al. proposed ctDNA can be used as a powerful tool for predicting MSS CRC patients’ response to regorafenib and nivolumab combination therapy (180). Gong et al. through four cases illustrated that ctDNA can be used as a dynamic predictive biomarker for colon cancer immunotherapy (128). In the CAVE trial, cetuximab and avelumab combination therapy were tested in RAS wild-type mCRC. In this study, patients’ KRAS, NRAS, BRAF, and EGFR-S492R mutation was analyzed through ctDNA, and the result show patients with RAS/BRAF WT ctDNA presented with better mOS and mPFS compared to patients with mutated ctDNA. These findings presented the potential role of ctDNA for colon cancer immunotherapy (181). However, the reports of ctDNA in the immunotherapy of colon cancer are still limited and ctDNA’s predictive role in immunotherapy needs to be verified by larger clinical studies. In addition, the following questions also need to be paid attention to in the further study of ctDNA (129, 182). First, the concentration of ctDNA in blood is relatively low. Thus, the sensitivity of the detection method is strictly required. Second, ctDNA is vulnerable to a variety of factors, such as trauma, which is a crucial factor affecting the determination of ctDNA. Therefore, strict avoidance of interfering factors is important for accurate measurement of ctDNA. Third, ctDNA is dynamically changing, thus the ctDNA results from different studies sampled at different points in time are difficult to unify and quantify, and there is yet to be a uniform detection standard for ctDNA. Furthermore, the testing method for blood ctDNA still needs to be optimized. Digital PCR (dPCR), amplification refractory mutation system (ARMS), and NGS are currently the main available methods for the detection of ctDNA. Each method has its strengths and limitations. dPCR with a low cost and relatively high sensitivity is the most used method for ctDNA detection. But the limitations of the dPCR are low throughput and the inability to detect unknown mutations. ARMS is moderate in cost, simple to operate, and can sequentially detect multiple mutations in a single gene, but it is not as sensitive as dPCR. The advantages of the NGS method lie mainly in the high throughput and sequencing of unknown mutations, but its economic cost is relatively high. Moreover, some study proposed the optimization of ctDNA detection (183). With the optimization of detection methods and the uniformization of standards, the value of ctDNA in immunotherapy of colon cancer will be better demonstrated.

In addition to ctDNA, various immune cells can also be tested in the peripheral blood, including T cells, B cells, NK cells, and myeloid cells (184). These circulating immune cells are proposed as predictive biomarkers for therapeutic response and clinical benefit of ICIs in solid cancer patients (185, 186). However, based on the existing study, the role of circulating immune cells in colon cancer immunotherapy is controversial. In a phase II study of durvalumab and tremelimumab with concurrent radiotherapy for pMMR mCRC patients, an increase in circulating CD8+T lymphocyte activation was observed in patients with an objective response. However, this combination of radiotherapy plus ICI did not meet the study endpoint criteria (187). In another study of mFOLFOX6 combined bevacizumab alone or with AdCEA vaccine combined avelumab immunotherapy for untreated mCRC, combination therapy generated brachyury-specific CD4+/CD8+T cells but did not improve patients’ PFS (130). In Cheng et al’s study, the peripheral blood of dMMR mCRC patients receiving anti-PD-1 immunotherapy was analyzed, the results show that the ratio of CD4+/CD8+ in peripheral blood and the frequency of CD4+ T cells are promising predictive biomarkers for dMMR mCRC patients responding to immunotherapy (131). According to Herting CJ et al’s study, the baseline levels and changes in circulating immunosuppressive myeloid and T cell subsets were not associated with advanced CRC patients’ response to pembrolizumab combined modified FOLFOX6 therapy (132). Moreover, according to Clouthier et al’s study, they found that the immune biomarkers were significantly varied between the blood and tissue (131). The reasons for this phenomenon are mainly related to the small sample size included in the existing studies and, similar to the reports of ctDNA, the different sampling times and analysis methods can also have an impact on the results. Test methods for the detection of various immune cell subsets including multi-color fluorescence flow cytometry, mass cytometry, and NGS, are still developing, and evaluation criteria need to be normalized (133). In addition, a larger population-based cohort study is necessary to further test the value of circulating tumor cells in immunotherapy of colon cancer.

Inflammation also plays an essential role in colon cancer tumorigenesis and influences patients’ immunotherapy response (188). Lymphocytes and neutrophils are two common indicators of the inflammatory state of the body in the peripheral blood (189). Some studies reported the predictive role of neutrophil-lymphocyte ratio (NLR) in colon cancer immunotherapy (134, 190, 191). In Saied et al’s study, they proposed that NLR changes correlated with CEA+ liver metastases CRC patients’ early responses to chimeric antigen receptor-modified T-cell (CAR-T) hepatic artery infusions (HAI) variations. Increased NLR levels were proven to be associated with poor responses following CAR-T HAI (190). The final results of the CAVE trial show that a baseline NLR <3 significantly correlated with improved survival of ctDNA RAS/BRAF WT patients after cetuximab plus avelumab therapy (191). Furthermore, through a retrospective study, Corti et al. proposed a blood-based biomarker, Pan-Immune-Inflammation Value which integrates neutrophil, platelet, monocyte, and lymphocyte counts, as a strong predictor of outcomes in MSI-H mCRC patients receiving ICIs (192). However, based on the limited number of studies conducted so far, the role of peripheral inflammatory cell-related involvement (e.g., NLR) in the immunotherapy of colon cancer remains to be further demonstrated.

In summary, tissue-based predictive biomarkers are more accurate and closely related to TME. However, there are still some challenges in some clinical situations. Tissue biopsies are invasive, and may be difficult to obtain in advanced and metastatic patients. In addition, intratumoral heterogeneity is prevalent among tumor tissues. Multipoint sampling is necessary to obtain more accurate results, but it is difficult to achieve in clinical settings, as it requires invasive procedures for the patient (193). Under such conditions, liquid biopsies through peripheral blood provide a method with minimally invasive, reproducible sampling and dynamically observe changes in indicators. The variety of information that can be acquired through liquid biopsy includes inflammatory cells, ctDNA, circulating immune cells, cytokines, and so on. Several peripheral blood biomarkers are now being proposed as predictive biomarkers for colon cancer immunotherapy response in existing clinical studies (**Table 1**). The development of high-throughput sequencing technology provides a deeper and broader view of peripheral blood biomarkers. However, since various multiplexed assays are employed for peripheral blood analyses, these assay protocols and their reporting methods need to be standardized, and additional studies will also be needed on the sampling time points, sensitivity, and specificity of each assay for clinical applicability. In further clinical studies, peripheral blood biomarkers will be developed as dynamic indicators for colon cancer immunotherapy.

## Conclusions and perspectives

In recent years, ICI-based immunotherapy has brought revolutionary breakthroughs in the treatment of colon cancer. While immunotherapeutic agents continue to be researched and developed, it is also worth focusing on precisely screening beneficial patients through predictive biomarkers. Currently, MSI is the only approved biomarker for screening colon cancer immunotherapy-benefiting patients. However, the results of existing clinical studies indicated the low efficacy of MSI as a predictive biomarker for colon cancer immunotherapy. Several new predictive biomarkers have been proposed in colon cancer immunotherapy. Developments have also been made in the detection method of predictive biomarkers for immunotherapy of colon cancer. In this review, we summarized the currently reported predictive biomarkers in existing studies of colon cancer immunotherapy.

**Figure 1:**
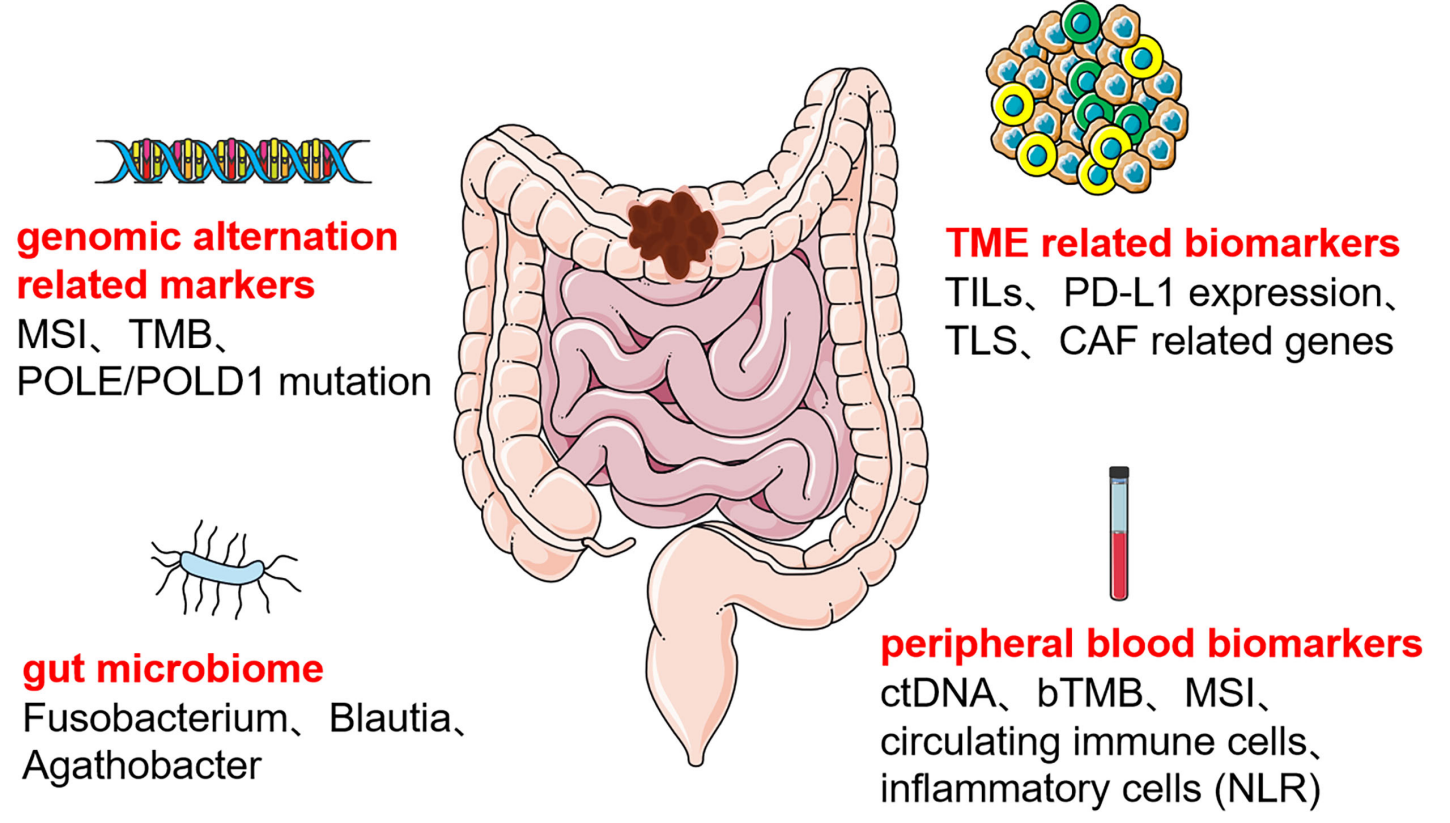
Predictive biomarkers of colon cancer immunotherapy MSI, microsatellite instability; TMB, total mutation burden; POLE:/POLD1, DNA polymerase ϵ (POLE) and δ (POLD1); TILs, tumor-infiltrating lymphocytes; PD-L1, programmed cell death-ligand 1; TLS, tertiary lymphoid structure; CAF, cancer-associated fibroblast; ctDNA, circulating tumor DNA; bTMB, blood total mutation burden; NLR, neutrophil-lymphocyte ratio.

We concluded that immunotherapeutic biomarkers reported in the clinical studies for colon cancer can be divided into four main categories (**Figure 1**), the first category of biomarkers related to genetic alterations, such as MSI, TMB, and POLE/POLD1; the second category of biomolecular markers related to TME, mainly included TILs, PD-L1 expression, TLS, and CAF related genes; the third category is certain specific gut microbiome; the fourth category is peripheral blood biomarkers, such as ctDNA, bTMB, circulating immune cells, and inflammatory cell related indicators. Based on currently reported clinical studies (**Table 1**), only MSI’s predictive role in colon cancer immunotherapy has been demonstrated in a larger cohort. The other immunotherapeutic biomarkers have only been reported in some small cohorts of colon cancer and are pending justification in larger cohorts. Some predictive biomarkers for colon cancer immunotherapy come from database analysis or retrospective studies, also waiting to be demonstrated by large cohort clinical studies. In addition, despite the attention given to the TME and gut microbiota in colon cancer immunotherapy, reliable biomarkers for colon cancer immunotherapy beneficial population selection are still lacking. Peripheral blood markers have been favored by many studies in recent years due to their non-invasive and multi-sampling advantages. However, a unified evaluation criterion is yet to be established. Furthermore, the testing methods of each biomarker are all waiting to be optimized to obtain more accurate testing results, and a unified judgment standard must be developed.

In summary, there is no optimal predictive biomarker for immunotherapy of colon cancer till now, and each biomarker has its limitations. Although the combined application of multi-methods for recognizing multi-indicators could improve the accuracy of biomarkers for colon cancer immunotherapy, large numbers of clinical trials are needed to verify that. With the optimization and improvement of the technology, more accurate biomarkers for predicting immunotherapy of colon cancer will help to stratify patients, which will also greatly improve the prognosis and the overall survival rate of patients

## Author contributions

(I) Conception and design: WH, CY, HZ; (II) Administrative support: CY; (III) Provision of study materials or patients: CY, HZ; (IV) Collection and assembly of data: WH, CY, HZ; (V) Manuscript writing: WH, CY, HZ. All authors contributed to the article and approved the submitted version.

## Funding

This study was supported by grants from the Key research projects of Science & Technology of Sichuan Province (2022YFS0189) and the Fundamental Research Funds for the Central Universities (2022SCU12058).

## Conflict of interest

The authors declare that the research was conducted in the absence of any commercial or financial relationships that could be construed as a potential conflict of interest.

## Publisher’s note

All claims expressed in this article are solely those of the authors and do not necessarily represent those of their affiliated organizations, or those of the publisher, the editors and the reviewers. Any product that may be evaluated in this article, or claim that may be made by its manufacturer, is not guaranteed or endorsed by the publisher.
